# Primary Spinal OPC Culture System from Adult Zebrafish to Study Oligodendrocyte Differentiation *In Vitro*

**DOI:** 10.3389/fncel.2017.00284

**Published:** 2017-09-14

**Authors:** Volker Kroehne, Vasiliki Tsata, Lara Marrone, Claudia Froeb, Susanne Reinhardt, Anne Gompf, Andreas Dahl, Jared Sterneckert, Michell M. Reimer

**Affiliations:** ^1^DFG-Center for Regenerative Therapies Dresden, Cluster of Excellence, Technische Universität Dresden Dresden, Germany; ^2^Deep Sequencing Group, Center for Molecular and Cellular Bioengineering (CMCB), Biotechnologisches Zentrum (BIOTEC), Technische Universität Dresden Dresden, Germany

**Keywords:** primary OPC culture, OPC, OL, spinal cord injury, oligodendrocyte progenitor cell, remyelination, adult zebrafish, human motor neuron

## Abstract

Endogenous oligodendrocyte progenitor cells (OPCs) are a promising target to improve functional recovery after spinal cord injury (SCI) by remyelinating denuded, and therefore vulnerable, axons. Demyelination is the result of a primary insult and secondary injury, leading to conduction blocks and long-term degeneration of the axons, which subsequently can lead to the loss of their neurons. In response to SCI, dormant OPCs can be activated and subsequently start to proliferate and differentiate into mature myelinating oligodendrocytes (OLs). Therefore, researchers strive to control OPC responses, and utilize small molecule screening approaches in order to identify mechanisms of OPC activation, proliferation, migration and differentiation. In zebrafish, OPCs remyelinate axons of the optic tract after lysophosphatidylcholine (LPC)-induced demyelination back to full thickness myelin sheaths. In contrast to zebrafish, mammalian OPCs are highly vulnerable to excitotoxic stress, a cause of secondary injury, and remyelination remains insufficient. Generally, injury induced remyelination leads to shorter internodes and thinner myelin sheaths in mammals. In this study, we show that myelin sheaths are lost early after a complete spinal transection injury, but are re-established within 14 days after lesion. We introduce a novel, easy-to-use, inexpensive and highly reproducible OPC culture system based on dormant spinal OPCs from adult zebrafish that enables *in vitro* analysis. Zebrafish OPCs are robust, can easily be purified with high viability and taken into cell culture. This method enables to examine why zebrafish OPCs remyelinate better than their mammalian counterparts, identify cell intrinsic responses, which could lead to pro-proliferating or pro-differentiating strategies, and to test small molecule approaches. In this methodology paper, we show efficient isolation of OPCs from adult zebrafish spinal cord and describe culture conditions that enable analysis up to 10 days *in vitro*. Finally, we demonstrate that zebrafish OPCs differentiate into Myelin Basic Protein (MBP)-expressing OLs when co-cultured with human motor neurons differentiated from induced pluripotent stem cells (iPSCs). This shows that the basic mechanisms of oligodendrocyte differentiation are conserved across species and that understanding the regulation of zebrafish OPCs can contribute to the development of new treatments to human diseases.

## Introduction

Remyelination is a well-known target to improve functional outcome after spinal cord injury (SCI) in humans. OLs are a prerequisite for saltatory conduction of action potentials along the axon and promote neuronal survival by providing metabolic support (Nave, 2010). In response to SCI, direct mechanical damage to tissue leads to loss of neurons, axons, and support cells, including mature myelinating OLs. Moreover, secondary injury by excitotoxicity and inflammatory processes results in a massive loss of OLs (Ma et al., 2016) - one of the most vulnerable cell types of the spinal cord. This leaves spared axons demyelinated, leading to a conduction block. This conduction block can be transiently removed by application of the potassium channel blocker 4-aminopyridine, resulting in a transient functional recovery in guinea pig and cat SCI models (Blight, 1989; Shi et al., 1997) and in humans (Grijalva et al., 2010). Promoting pro-remyelinating strategies will support axons permanently and thus improve functional long-term recovery.

Several clinical trials have been performed using myelinating cells, including autologous olfactory ensheathing cells and Schwann cells, with results demonstrating an improvement in electrophysiological tests and functional assessment (Chen et al., 2014). The caveat is: harvesting, propagating and transplanting autologous remyelinating cells involves transplantation-associated technical challenges and risks. These issues could be circumvented if the remyelinating capacity of endogenous OPCs was increased to provide sufficient remyelination. In addition, pro-remyelinating strategies would also be beneficial for newly generated axons to ensure long-term stability. Until recently, axonal regrowth was thought to be unlikely, but current studies found several promising approaches to how axonal regeneration across the lesion site could be achieved (Ruschel et al., 2015).

Therefore, several research groups attempt to control endogenous OPC behavior using small molecule-based strategies, some of which have proven to be quite successful: e.g., by using OPCs derived from mouse pluripotent EpiSC lines (Najm et al., 2015). However, these approaches do not take into account the age-status of dormant OPCs *in vivo*.

The use of OPC cultures is one of the corner stones in elucidating and testing novel targets for pro-remyelinating strategies. Nevertheless, using human iPSC derived OPCs is still difficult due to the lack of efficient protocols to generate OPCs and OLs (Goldman and Kuypers, 2015; Ehrlich et al., 2017). Additionally, OPCs display a regional identity which leads to different myelination phenotypes, like e.g., myelin sheath length (Bechler et al., 2015). However, mouse pluripotent EpiSC lines (Najm et al., 2015) are generated in vast numbers and allow rapid high-throughput small molecule screening but, similar to human iPSC derived OPCs, do not show spinal region specific OPC subpopulations. A further drawback is that induced OPCs never enter the dormant stage they take on *in vivo*, thereby making identification of OPC activating modulators difficult.

Interestingly, zebrafish OPCs are able to generate full thickness myelin sheaths after demyelination of the optic nerve (Munzel et al., 2014). This is congruent with the observation that the zebrafish CNS has a remarkable ability to functionally regenerate after injury (Becker et al., 2004; Reimer et al., 2008, 2009, 2013; Kroehne et al., 2011; Barreiro-Iglesias et al., 2015).

**FIGURE 1:**
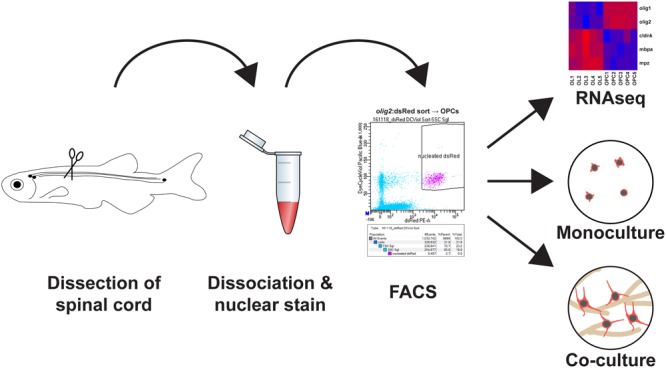
Main steps to obtain a highly pure adult zebrafish spinal oligodendrocyte progenitor cell (OPC) population: (1) Adult zebrafish spinal cord tissue is dissected. (2) The tissue is first dissociated by enzymatic digestion and then incubated with a nuclear dye. (3) The cell suspension is FACsorted based on cytoplasmic and nuclear fluorescence. (4) The purified OPCs can then be used directly for transcriptome analysis, cell culture as monoculture to study dormant OPCs or in motor neuron/OPC co-cultures to study oligodendrocyte differentiation.

In this methodology paper, we describe how zebrafish spinal OPCs can be isolated and used in cell culture to test underlying pathways that could explain their resilience and remyelinating abilities (**Figure 1**).

## Materials and Methods

### Animals

Fish are kept and bred in our fish facility according to standard methods (Westerfield, 1989). All experiments were performed in compliance with animal welfare legislation. Procedures were approved by the ethical approval committee of the Regierungspräsidium Dresden, Germany: TVV 53/2015 and TV 9/2016.

All efforts were made to minimize animal suffering and the number of animals used. We used Tg(*olig2*:dsRed2) (Kucenas et al., 2008), Tg(*olig2*:eGFP) (Shin et al., 2003) and Tg(*mbp*:eGFP) (Jung et al., 2010).

### Spinal Cord Lesion

As described previously (Becker et al., 2004), fish were anesthetized by immersion in 0.033% aminobenzoic acid ethylmethylester (MS222; Sigma) in PBS for 5 min. A longitudinal incision was made at the side of the fish, and the spinal cord was completely transected under visual control 4 mm caudal to the brainstem–spinal-cord junction.

### Spinal Cord Dissection, Tissue Dissociation and Nuclear Labeling

Adult zebrafish were terminally anesthetized, their spinal cords exposed and the spinal cord tissue was carefully removed. Up to five spinal cords were dissected at once and placed in 1 mL of Hanks′ Buffered Salt Solution (HBSS, Gibco). For the dissociation of spinal cords into a single cell suspension, the tissue was incubated for 3 min with 100 μL 0.25% Trypsin (Sigma; T4549)/1 mM EDTA at room temperature and triturated using a 200 μL pipette during the whole incubation time avoiding the formation of air bubbles. Then, tissue digestion was stopped by adding 100 μL of 20% fetal calf serum (Sigma; F0804) in 2 mM CaCl_2_ and 300 μL HBSS. Afterward the cell suspension was allowed to sit for 5 min on ice, before it was applied to a 20 μm (OPCs, Miltenyi Biotec, # 130-101-812) or a 35 μm (OL, Corning, # 352235) cell strainer, respectively. After washing with 9 mL of HBSS, the cells were pelleted by centrifugation for 5 min at RT at 300 g. The supernatant was removed, cells were resuspended in 1 mL HBSS including 1 μL Vybrant^TM^ DyeCycle^TM^ Violet Stain (Molecular Probes, Invitrogen, V35003) and incubated for 30 min at 28°C to stain cellular nuclei.

### FACsorting of Myelin Rich Spinal Cord Tissue

Cell suspensions were sorted directly into 96 well plates, filled with culture medium only, or pre-seeded with motor neurons (MNs) using a BD FACSAria sorter. For the detection of GFP a 488 nm excitation laser and a 530/30 bandpass filter were used. DsRed was detected with a 582/15 bandpass filter after excitation with a 561 nm laser. Vybrant^TM^ DyeCycle^TM^ Violet Stain was detected after 405 nm excitation and a 450/40 bandpass filter. Cellular events were defined by forward and side scatter profile and by the dsRed or GFP signal of the corresponding transgenic line. From this selection, all events that showed incorporation of the Vybrant^TM^ DyeCycle^TM^ Violet Stain were gated because this proved to be an ideal selection to separate the small zebrafish CNS cells from remaining cellular and myelin debris. To assess purity and viability of the sorted cells post-sort analysis of the purified cells after addition of Propidium Iodide at a final concentration of 1 μg/mL was performed.

### Cell Culture Conditions in OPC Monocultures

Two days before dissection, the wells of a 96-well plate (Greiner Bio One, # 655090) were coated with 10 μg/mL Poly-D-Lysine solution (Merck Millipore, # A-003-E) in DEPC H_2_0 and left under a biosafety cabinet overnight (50 μL/well).

The next day, Poly-D-Lysine was removed and wells were washed three times with DEPC H_2_0 to remove unbound Poly-D-Lysine. After air-drying, 10 μg/mL of laminin solution (Sigma Aldrich, # L2020) in sterile PBS was added to the wells (50 μL/well) and the plate was left at 37°C overnight. On the day of the dissection, laminin solution was removed and the wells were washed twice with sterile PBS and once with DEPC H_2_0 to remove unbound coating solution. After air-drying, 280 μL of Leibovitz’s L-15 Medium (Gibco, # 11415-049) supplemented with 15% FBS (Gibco, Performance Plus United States, # 1000-0-044), 1% Glutamax (Gibco, # 350-050-038) and 1% penicillin/streptomycin (Gibco, # 15-140-122) was added to each well. The plate was left at 28°C until cell seeding. Fluorescently sorted OPCs were plated at a density of 10000 cells per well and left at 28°C for a maximum of 10 days. Culture medium was exchanged every 2 days, starting at 3 days in culture.

### Cell Culture Conditions in OPC/MN Co-cultures

The complete human iPSC culture and differentiation protocol for MN generation is described in detail elsewhere (Reinhardt et al., 2013). Briefly, a population of expandable neural progenitors (NPCs) is obtained from iPSCs via dual SMAD inhibition. Using a defined cocktail of small molecules, NPCs can subsequently be differentiated into MNs. For MN plating, cells were detached in their early maturation stage, i.e., day 3, using Accutase (Sigma), and distributed on Matrigel (Corning)-coated plates in MN maturation medium, consisting of N2B27 medium supplemented with 2 ng/mL BDNF (Peprotech), 2 ng/mL GDNF (Peprotech), 1 ng/mL TGF-β3 (Peprotech), 200 μM ascorbic acid, and 100 μM dbcAMP. Y-27632 (Sigma) was included in the first day to increase the chances of MN survival upon re-plating. N2B27 medium consisted of DMEM-F12/Neurobasal medium 1:1, 0.5% N2 supplement, 1% B27 supplement lacking vitamin A, and 1% PS/G (all Thermo Fisher Scientific). For plate coating, the Matrigel mixture was diluted to 1:100 in ice-cold Knockout DMEM (Invitrogen) prior to overnight coating at RT. For long-term storage, coated plates were wrapped with Parafilm and kept in the fridge for up to 1 month. Adult zebrafish OPCs were FACsorted on top of the MN culture at day 4–7 of differentiation and cultured with MN maturation medium at 28°C under 5% CO_2_. Medium was replaced every second day.

### Immunofluorescence

Immunohistochemistry on paraformaldehyde-fixed spinal cord sections (50 μm thickness) has been described previously (Reimer et al., 2008). Cells were fixed in 4% paraformaldehyde (Agar Scientific, 16% EM Grade, Methanol free, R1026) diluted in PBS for 10 min at room temperature, washed gently with 0.1% Triton X-100 (Sigma Aldrich, # T8787) in PBS and incubated with primary antibodies in 0.1% Triton X-100 in PBS for 1 h at room temperature.

After washing with 0.1% Triton X-100 in PBS, cells were stained with secondary antibodies in the same solution for 2 h at room temperature. Cells were finally stained with 0.3 μM DAPI (Roth, # 6335.1) in PBS for 10 min at room temperature, washed in PBS and processed accordingly.

### Antibodies

Primary antibodies used were: mouse monoclonal against Glial Fibrillary Acidic Protein (GFAP, 1:250, Clone GA5, Merck Millipore, MAB3402), rabbit polyclonal against SRY-box containing gene 10 (Sox10, 1:250 (cultures) and 1:500 (sections), Biozol GTX128374-200) and a custom-made rabbit polyclonal against Myelin Basic Protein [MBP, 1:75, (Pinzon-Olejua et al., 2017) a kind gift from Michael Brand, CRTD, TU Dresden, Germany].

Secondary antibodies used were goat anti-Mouse IgG Alexa Fluor^®^555, (Molecular Probes, Invitrogen, A21424) and donkey anti-Rabbit IgG Alexa Fluor^®^647, (Jackson ImmunoResearch Europe Ltd., 711-605-152), both used at a 1:750 dilution.

### Data Acquisition and Processing

Confocal images of cell cultures were acquired with a Zeiss LSM 780/FCS inverse microscope using an LCI PlnN 63 × /1.3 NA W DICIII objective in a tile mode (10 × 10 tiles, 10% overlaps). Tissue sections were imaged with a Leica TCS-SP5 confocal microscope using HC PL APO CS 20 × /0.7 NA, HCX PL APO 40 × /1.25 NA and HCX PL APO 63 × /1.2 NA objectives. To minimize channel crosstalk, single channel images were acquired sequentially. Stacks of overview images were maximum projected using Fiji. Co-localization of markers was verified in single confocal stacks using the 63× objectives. Adobe Photoshop CS6 was used to adjust brightness and contrast. Illustrator CS6 was used to compile the figures.

### Data Quantification and Statistical Analysis

Cells were manually counted with the Cell Counter Plugin of Fiji. For quantification of total cell numbers, a representative area corresponding to ∼5% of the total well area were quantified and for each time-point, 3–4 wells were counted. Microsoft Excel was used to process the measured data. To analyze significance, *P*-values were determined with GraphPad Prism using One Way ANOVA with Dunns *Post hoc* test. Error bars represent SEM. *P* > 0.05 was not considered significant.

### Deep Sequencing and Data Analysis

For the RNAseq experiment, the SMARTer Ultra Low Input RNA for Illumina Sequencing Kit - HV from Clontech was used to reverse transcribe the RNA and amplify full-length cDNA according to the user manual. Therefore, 2000 cells from five biological replicates of both lines Tg(*olig2*:eGFP) and Tg(*mbp*:eGFP) were directly FACsorted in 5 μL reaction buffer containing RNAse Inhibitor. After amplification of cDNA using 12 cycles, the cDNA was sheared to 200 bp fragment length with 8-micro TUBE strip in the Ultrasonicator Covaris LE220, followed by a library prep using NEBNext Ultra DNA Library Prep Kit for Illumina (NEB). The libraries were sequenced with 75 bp single end on a Hiseq2500 with a minimal sequencing depth of 27 million reads and mapped to GRCz10 with GSNAP (v 2016-09-23). Transcripts were counted with feature Counts v 1.5.2 based on Ensembl version 81. The gene expression values were normalized by the R-package DESeq2 in R (v 3.3.0).

### Accession Numbers

The next generation sequencing datasets generated in this study have been submitted to GEO and are publically available under accession number GSE100821.

## Results

### Rapid De- and Remyelination in Response to Spinal Cord Injury

**FIGURE 2:**
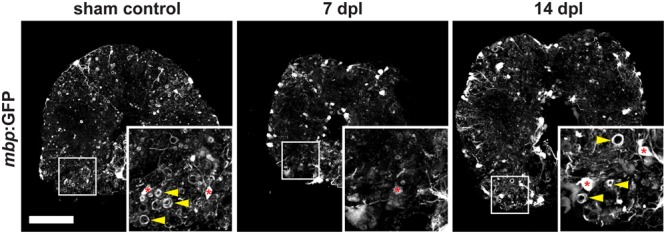
Severe demyelination and loss of oligodendrocytes (red asterisks) occur until 7 days post spinal transection lesion (dpl) in the adult zebrafish spinal cord close to the site of injury. In contrast, in sham-lesioned control spinal cords many *mbp*:GFP-positive myelin sheaths are found (yellow arrowheads). At 14 dpl, a partial re-establishment of the constitutive myelination pattern is observed (yellow arrowheads). 50 μm cross section; distance to lesion site ≤ 350 μm; scale bar represents 100 μm.

A complete spinal cord transection in adult zebrafish (Becker et al., 2004; Reimer et al., 2008) causes full paralysis caudal to the site of injury. In order to analyze the fate of mature oligodendrocytes (OLs) at the lesion site after SCI, we transected the spinal cord of transgenic Tg(*mbp*:GFP) zebrafish. In this reporter line OLs are labeled by GFP, enabling the identification of myelin sheaths as tube-like structures highlighted by GFP (**Figure 2**). We find that in response to SCI, myelin sheaths and OL cell bodies are lost at 7 days post lesion (dpl) compared to sham controls (**Figure 2**). At 14 dpl, a remyelination response has been initiated and newly formed myelin sheaths, as well as an increase in the number of OL cell bodies, are observed (**Figure 2**).

### Oligodendrocyte Progenitor Cells in the Adult Zebrafish Spinal Cord

**FIGURE 3:**
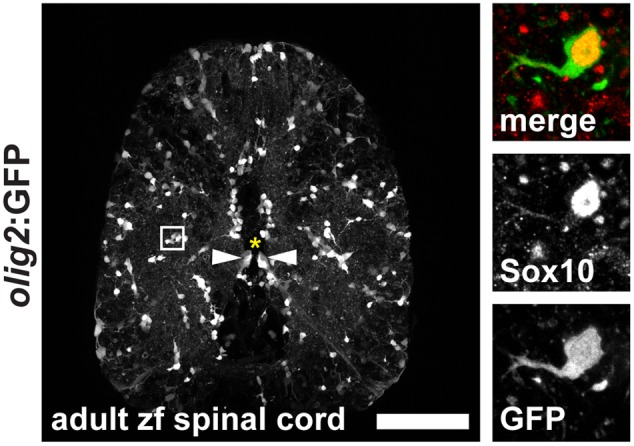
Expression of *olig2*:GFP in the adult zebrafish spinal cord marks OPCs in the parenchyma and a subpopulation of ventral radial glia at the central canal. OPCs are evenly distributed throughout the parenchyma in an unlesioned zebrafish spinal cord. Inset: OPCs display short arbors and co-express Sox10. 50 μm cross section; asterisk marks the central canal; scale bar represents 100 μm.

During development of the vertebrate CNS mature OLs form from OPCs that derive from pMN progenitor cells of the neural tube (Park et al., 2002; Shin et al., 2003; Ravanelli and Appel, 2015). In the adult brain and spinal cord OPCs are maintained and function as a constitutive progenitor pool for the formation of new OLs (Park et al., 2007). In zebrafish, OPCs are marked by high expression of *olig2*:GFP, a pan-vertebrate oligodendroglial lineage marker (Czopka and Lyons, 2011), and are evenly distributed throughout the white and gray matter of the adult spinal cord parenchyma (**Figure 3**). OPCs co-express the transcription factor Sox10, a neural crest, as well as oligodendroglia lineage marker in the CNS of vertebrates (**Figure 3** and **Supplementary Figure S1**). A subpopulation of ventral radial glia also expresses *olig2*:GFP. Radial glia are identified by ventricular cell bodies (arrowheads) touching the central canal (asterisk) and radial processes spanning the entire ventral spinal cord. *Olig2*:GFP-positive OPCs can be easily distinguished from mature *mbp*:GFP-expressing OLs by their specific morphology displaying short arbors and the lack of GFP-positive myelin sheaths (**Figures 2, 3**).

### Generation of Spinal OPC Preparations with High Purity and Vitality

**FIGURE 4:**
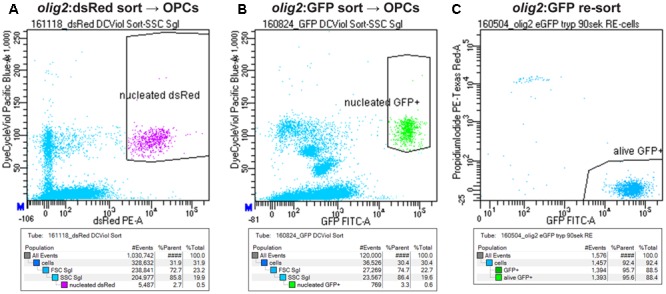
Strategy to sort OPCs using FACS. **(A)** Gating of OPCs based on *olig2*:dsRed and nuclear counter stain signals. **(B)** Gating of OPCs based on *olig2*:GFP and nuclear counter stain signals. **(C)** Re-sort of FACsorted *olig2*:GFP cells, counterstained with Propidium iodide to detect dead cells, shows a purity and viability of >95% of the sorted population.

Zebrafish embryos are a standard model organism in developmental biology. Because of their small size and transparency, they offer ideal conditions for functional analysis: e.g., straightforward application of small molecules just by adding the compounds to the water. Additionally, live imaging can easily be done up to several days after fertilization. In contrast to embryos, functional analysis of cells in the adult zebrafish spinal cord is much more challenging. Therefore, we established a platform to analyze zebrafish OPCs *in vitro*. We developed a streamlined and fast, though inexpensive, protocol that allows direct access to a pure and vital population of zebrafish OPCs in less than 2 h. This simple protocol is based on Trypsin digestion of dissected fish spinal cord, followed by addition of the Vybrant DyeCycle Violet Stain as a nuclear marker of living cells for fluorescent activated cell sorting (FACS) of OPCs. Cellular events were defined by forward and side scatter profile and by fluorescent marker (either dsRed or GFP) signal of the corresponding transgenic line. From this selection, all events that showed incorporation of the Vybrant DyeCycle Violet Stain were gated because this proved to be an ideal selection to separate the small zebrafish CNS cells from remaining cellular, i.e., mostly myelin debris (small cells cannot be separated from all cellular debris when only using forward and side scatter, **Figures 4A,B**). To assess purity and viability of the sorted cells we performed a post-sort analysis of the purified cells after addition of Propidium Iodide to stain non-viable cells. Less than 5% of cellular events corresponded to dead cells showing uptake of Propidium Iodide (**Figure 4C**). More than 95% of the sorted cells showed the fluorescent lineage marker, demonstrating high purity of sorted cell populations (**Figure 4C**). Typically, approximately 30000 cells could be sorted using five adult spinal cords as starting material.

### RNAseq Analysis Shows a Clear Separation of OPC and OL Transcriptomes

**FIGURE 5:**
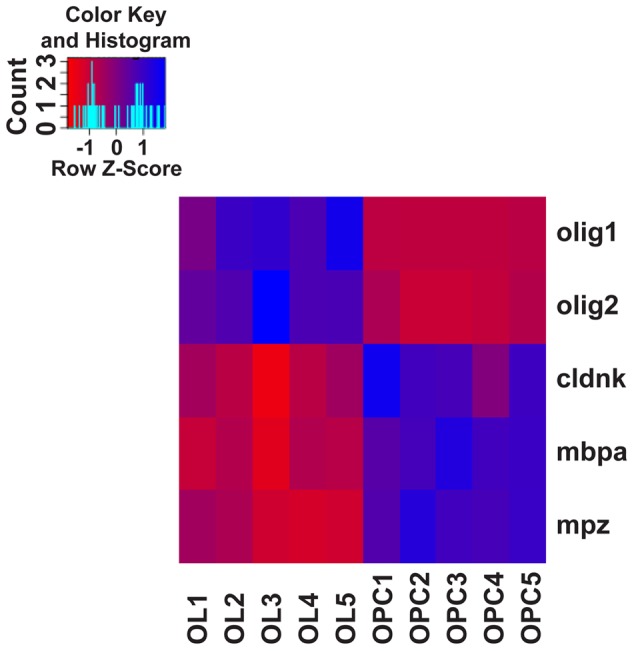
Heat-map of canonical oligodendroglial marker gene expression. Assessing the gene expression levels of well-described differentiation markers of the oligodendroglial lineage from transcriptome/RNAseq data confirms the identity and differentiation status of FACsorted cell populations: Based on the expression of the oligodendrocyte lineage markers *olig1* and *olig2* and the mature OL markers *cldnk, mbpa* and *mpz*, biological replicates of OPC and OL populations are clearly separated. Gene expression levels of the chosen genes are standardized by z-transformation allowing the direct comparison of up- and downregulation.

To corroborate the identity and differentiation status of our FACsorted populations, we carried out gene expression analysis using RNAseq of sorted *olig2*:GFP- (OPCs) and *mbp*:GFP-positive cells (OLs, **Supplementary Figure S2**) and transcriptomes were analyzed for differential gene expression of well-known markers of the oligodendroglial lineage (**Figure 5**). A high degree of cell type specificity was revealed: OPC and OL populations are distinctly separated, as shown by expression profiling for the mature OL/myelin marker genes *cldnk* (Claudink), *mbpa* (Myelin Basic Protein) and *mpz* (Myelin Protein Zero). Interestingly, the expression levels of the oligodendroglial lineage markers *olig1* and *olig2* are much higher in the OPC population than in the mature OL population.

### OPC Cultures Are Stable for More Than 10 days *In Vitro*

**FIGURE 6:**
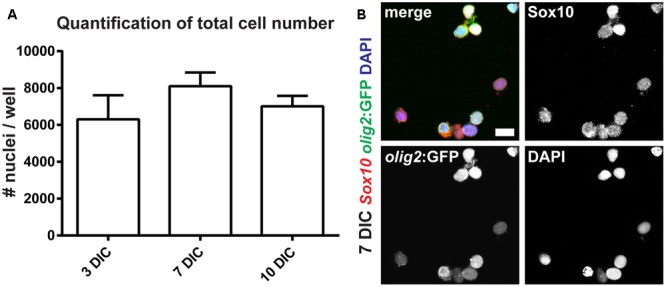
Oligodendrocyte progenitor cells can be maintained as monocultures. **(A)** OPC numbers are stable over the course of 10 days in culture (DIC). **(B)** The vast majority of *olig2*:GFP cells co-express Sox10, identifying them as OPCs; scale bar represents 10 μm.

On the one hand, the protocol described above can be used to establish acute cultures of OPCs for immediate cellular analyses *ex vivo*, like, e.g., electrophysiological measurements of freshly isolated OPCs and OLs. On the other hand, we also wanted to analyze OPC fate over longer culture periods *in vitro*. Specifically, we were interested in the robustness of the cultures and in their differentiation capacity. Therefore, we first quantified the number of cells over different time points. At 3, 7, and 10 days in culture (DIC) cell numbers do not change significantly, indicating stability of the culture and suggesting that OPCs neither undergo significant cell death, nor are activated toward overt proliferation under these conditions (**Figure 6A**). However, at 14 DIC we observed a significant detachment of cells from the bottom of the culture plates, as well as an increase in the number of dying cells (weak DAPI staining, pyknotic nuclei and weak cytoplasmic signal, data not shown).

The morphology of cells at 7 DIC is round with short processes, and significant differentiation of OPCs toward OLs is not apparent (**Figure 6B**). To confirm cell identity and differentiation status, we performed staining of cultures using antibodies against GFAP (radial glia marker), Sox10 (oligodendroglial lineage marker) and MBP (marker of mature OL) at different time points *in vitro*. At 3 DIC we analyzed 1052 *olig2*:GFP-positive cells: all cells co-expressed Sox10, and none of them expressed GFAP, identifying them as OPCs. At 7 DIC we analyzed 749 cells, of which 745 co-expressed Sox10 (**Figure 6B**). At 10 DIC, we analyzed 322 cells for MBP expression. We failed to detect any cell expressing MBP, showing that under these conditions OPCs do not spontaneously differentiate into mature OLs.

### Adult Zebrafish OPCs Differentiate into MBP-Positive Mature OLs in a Human Motor Neuron Co-culture Model

**FIGURE 7:**
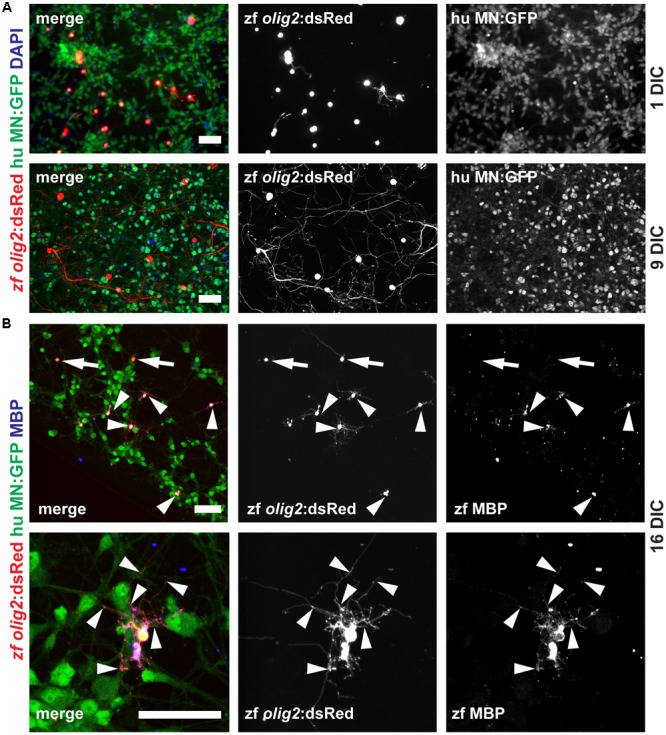
Adult zebrafish OPCs differentiate into MBP-positive mature OLs in human MN co-cultures. **(A)** OPCs differentiate over time in co-culture as identified by increased branching of the seeded OPCs. **(B)** At 16 DIC, the majority of oligodendroglial cells expresses the mature OL/myelin sheath marker protein MBP (arrowheads) while only a few cells fail to differentiate and remain MBP-negative (arrows); scale bars represent 50 μm.

The overall goal of this project was to establish an *in vitro* myelination model based on adult zebrafish OPCs. Since we did not detect formation of myelin in our monotypic cultures we reasoned that a co-culture model including motor neurons (MNs) could drive OPCs into OL differentiation and hence promote myelination. First, we determined if the ratio of OPCs vs. MNs is influencing OPC differentiation. We cultured 5000 OPCs together with 0, 10000, 20000, 40000, 80000, and 160000 MN per well (**Supplementary Figure S3**). We find a correlation of OPC differentiation state and MN numbers based on OPC morphology at 1 DIC. The number of OPCs that start differentiating, i.e., cells with long and branched processes, is increased in cultures with higher MN numbers (**Figure 7A** and **Supplementary Figure S3**). On the contrary, almost all cells in cultures without MNs have a round morphology (**Supplementary Figure S3**). The degree of branching and the length of OPC processes is strongly increased with culture time, showing that OPCs continue to differentiate toward mature OL (**Figure 7A** and **Supplementary Figure S4**). At 16 DIC we analyzed the expression of MBP protein in the cultures by antibody staining. Indeed, we find many cells that are positive for MBP (**Figure 7B**), showing that OPCs efficiently differentiated into highly branched OLs.

## Discussion

Oligodendrocyte progenitor cells in the adult brain and spinal cord are not readily accessible and functional analysis or live imaging are very challenging tasks *in vivo*. Therefore, we decided to establish a novel *in vitro* platform that would allow us to study adult zebrafish OPC proliferation, migration, differentiation and myelination, combined with the possibility to perform live imaging, e.g., in the context of OPC migration studies (O’Meara et al., 2016). Furthermore, this method can be used to generate cultures for electrophysiological measurements.

### *In Vitro* Models of OPC Proliferation, Migration, Differentiation and Myelination

Studies using isolated primary OPCs have been carried out for almost 4 decades (McCarthy and de Vellis, 1980). However, most isolation protocols start with mixed cultures from embryonic or neonatal tissue from rat. The mixed cultures can be enriched for OPCs by different methods, the most popular and simple being the shake-off technique that is based on differential adhesive properties of glial populations, reviewed in (Barateiro and Fernandes, 2014). The enrichment and survival of rat OPCs is largely dependent on the presence of growth factors, like basic fibroblast growth factor (FGF-2) and platelet derived growth factor (PDGF), the cell seeding density and also the composition of the basal medium used (Kleinsimlinghaus et al., 2013). The establishment of mouse OPC cultures is much more difficult than from rat, mostly because of the lack of surface markers and the tendency of mouse OPCs to spontaneously differentiate into other lineages (Chen et al., 2007; Zhu et al., 2014). Recently, a new method to enrich OPCs in mixed cultures from mouse cortices has been developed. Using a cocktail of the growth factors Platelet Derived Growth Factor-AA (PDGFaa), basic fibroblast growth factor (bFGF) and epidermal growth factor (EGF) OPCs could be propagated (Yang et al., 2016). Interestingly, in mouse the efficiency of the OPC isolation is strongly correlated with the age of the donor tissue: the number of adult OPCs reaches 12% of the cell number that could be isolated from neonates (Pillai and Ross, 1986). Human OPCs have been isolated from different sources, like cerebral cortex (Pillai and Ross, 1986), generated from fetal neurospheres (Lu et al., 2015) and very recently also derived from iPS cells (Ehrlich et al., 2017; Rodrigues et al., 2017). Generally, human OPCs seem to be a difficult model to work with because of their slow growth and differentiation behavior.

### Studying OPCs from the Adult Zebrafish Spinal Cord

Elucidating the underlying mechanisms of successful remyelination after SCI in the regenerating model organism zebrafish can be highly beneficial to develop pro-remyelinating strategies for improving endogenous remyelination in patients with SCI. Adult zebrafish recover functionally within 6 weeks after a full spinal cord transection. Lost neurons are replaced, axons regrow and remyelination of the demyelinated spinal cord and the newly generated axons bridging the lesion site occur (Becker et al., 1997; Reimer et al., 2008). However, the underlying mechanisms of the superior remyelination capacity in zebrafish (Munzel et al., 2014) are not understood. Investigating the OPC intrinsic mechanisms of dormancy, activation and remyelination in an *in vitro* system is a promising approach. The advantage of adult spinal cord FACsorted OPCs lies in the region identity and age-status of these progenitor cells, information that is not available from iPSC-derived OPCs or the other models described above. Additionally, sorted zebrafish OPCs have entered dormancy *in vivo*, which also allows studying their activation. In this study we could show that OPCs sorted from spinal cord tissue can be successfully taken into culture and that these monocultures are viable for at least 10 days. Cells cultured under these conditions resemble a dormant adult spinal OPC population of an uninjured spinal cord. In order to study activation and differentiation, we propose a novel co-culture system with human iPSC-derived motor neurons (Reinhardt et al., 2013). Although rat/mouse myelinating xenocultures have been described before (O’Meara et al., 2011), the use of fish/human co-cultures to our knowledge has not been reported. The use of zebrafish OPCs and human motor neurons enables the identification of evolutionary conserved signaling pathways. This increases the chances of utilizing the obtained results in a translational framework.

## Author Contributions

VK, VT, and MMR contributed to the design of the work, the acquisition, and interpretation of the data. VK and VT performed dissections, cell culture, tissue vibratome-sectioning, immunohistochemistry, confocal imaging; CF supervised, maintained, and genotyped the zebrafish colony and performed tissue dissociation. LM and JS contributed the motor neuron cell cultures for co-culture experiments. AG performed FACS and Flow Cytometry Analysis, SR and AD performed the deep sequencing and bioinformatic analysis. VK compiled the figures. MMR and VK co-wrote the manuscript; all experiments were performed in the MMR laboratory.

## Conflict of Interest Statement

The authors declare that the research was conducted in the absence of any commercial or financial relationships that could be construed as a potential conflict of interest.

## Supplementary Material

FIGURE S1 Overlap of *olig2*:GFP expression and nuclear Sox10 antibody staining in the adult zebrafish spinal cord. Almost all *olig2*:GFP-expressing cells in the parenchyma show a nuclear signal for Sox10 (arrowheads). Also GFP-negative longitudinal structures in the parenchyma and along the midline dorsal to the central canal (asterisk) show a signal for Sox10. These structures could represent axonal processes (from sensory neurons: dorsal root and sympathetic ganglia) and meninges-like membranes. 50 μm cross section; scale bar represents 100 μm.

FIGURE S2 Strategy to sort OLs using FACS. Gating of OLs based on *mbp*:GFP and nuclear counter stain signals.

FIGURE S3 Morphology of OPCs varies with different numbers of MNs. Without the addition of MNs, most OPCs have a small and round morphology at 1 DIC. The number and branching complexity of OPC processes increase when MNs are added and are highest at the highest MN density of 160000 cells; scale bar represents 50 μm.

FIGURE S4 After longer culture periods OPCs adopt the morphology of mature OLs in OPC/MN co-cultures. At 7 DIC OPC processes increase in size, length and complexity of branching compared to 2 DIC. At 7 DIC the proximal region of the processes is enlarged compared to more distal regions (arrowhead). In the central part of some processes close association of axon and oligodendroglial processes can be seen (arrowheads); scale bar represents 50 μm.
