# Supplementary figures and images for: Primary Spinal OPC Culture System from Adult Zebrafish to Study Oligodendrocyte Differentiation In Vitro
Source: Front Cell Neurosci. 2017 Sep 14;11:284. doi: 10.3389/fncel.2017.00284 (PMC5603699; doi:10.3389/fncel.2017.00284)

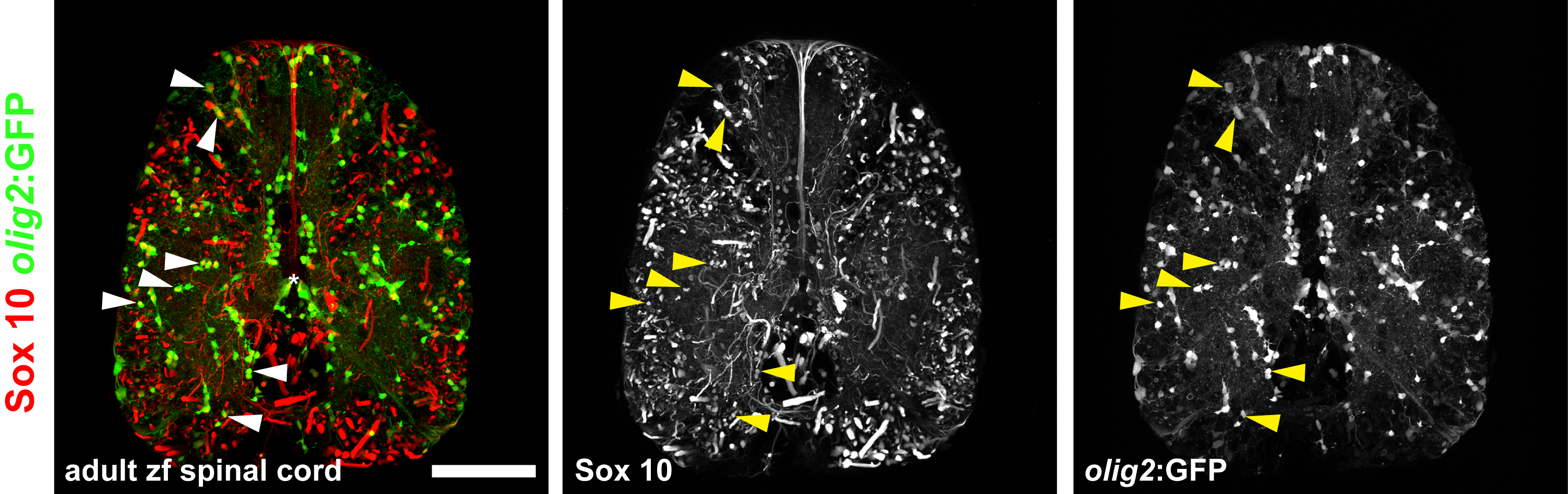

Supplement: FIGURE S1 — Overlap of olig2:GFP expression and nuclear Sox10 antibody staining in the adult zebrafish spinal cord. Almost all olig2:GFP-expressing cells in the parenchyma show a nuclear signal for Sox10 (arrowheads). Also GFP-negative longitudinal structures in the parenchyma and along the midline dorsal to the central canal (asterisk) show a signal for Sox10. These structures could represent axonal processes (from sensory neurons: dorsal root and sympathetic ganglia) and meninges-like membranes. 50 μm cross section; scale bar represents 100 μm. [file Image_1.TIF]

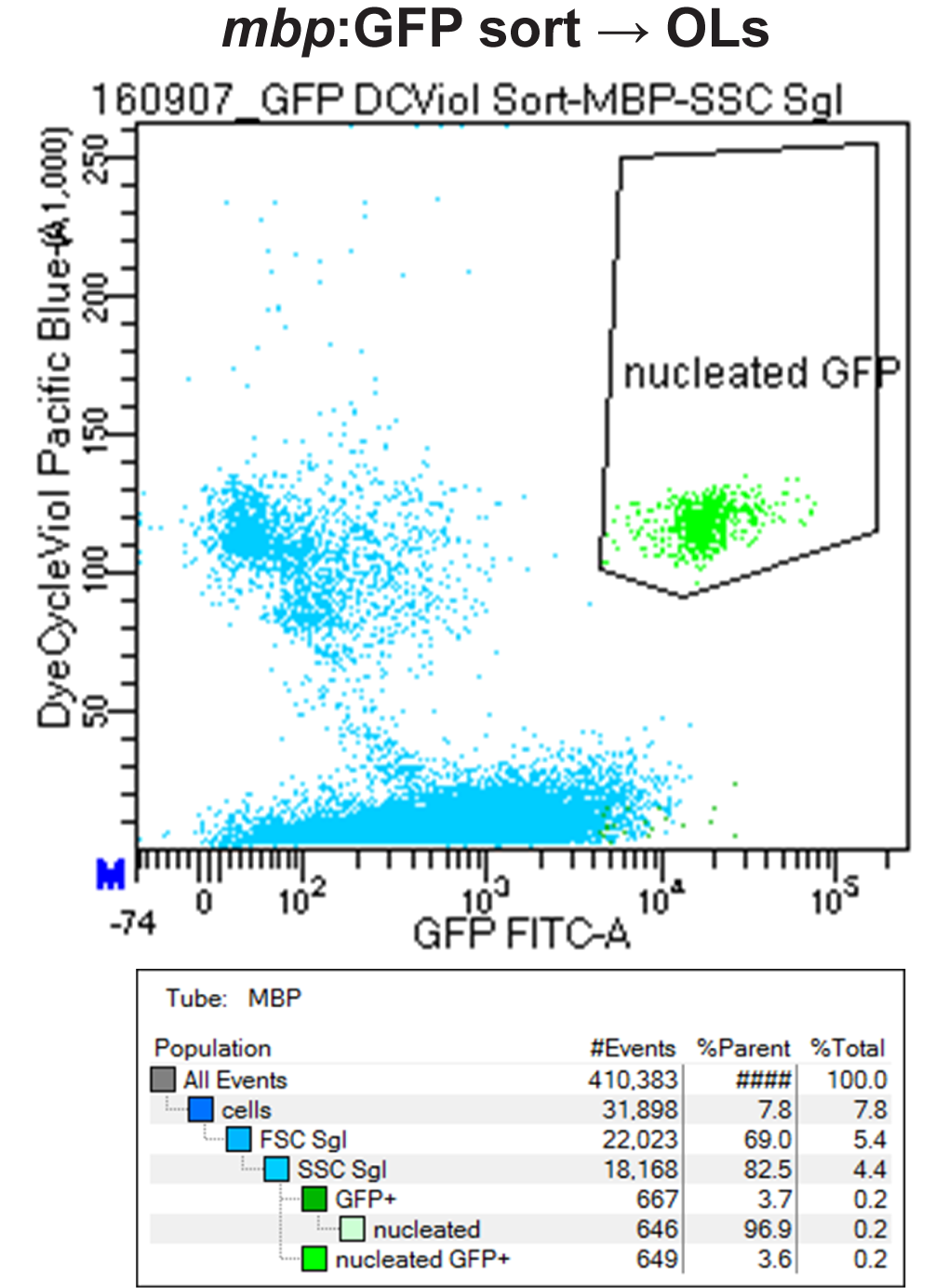

Supplement: FIGURE S2 — Strategy to sort OLs using FACS. Gating of OLs based on mbp:GFP and nuclear counter stain signals. [file Image_2.TIF]

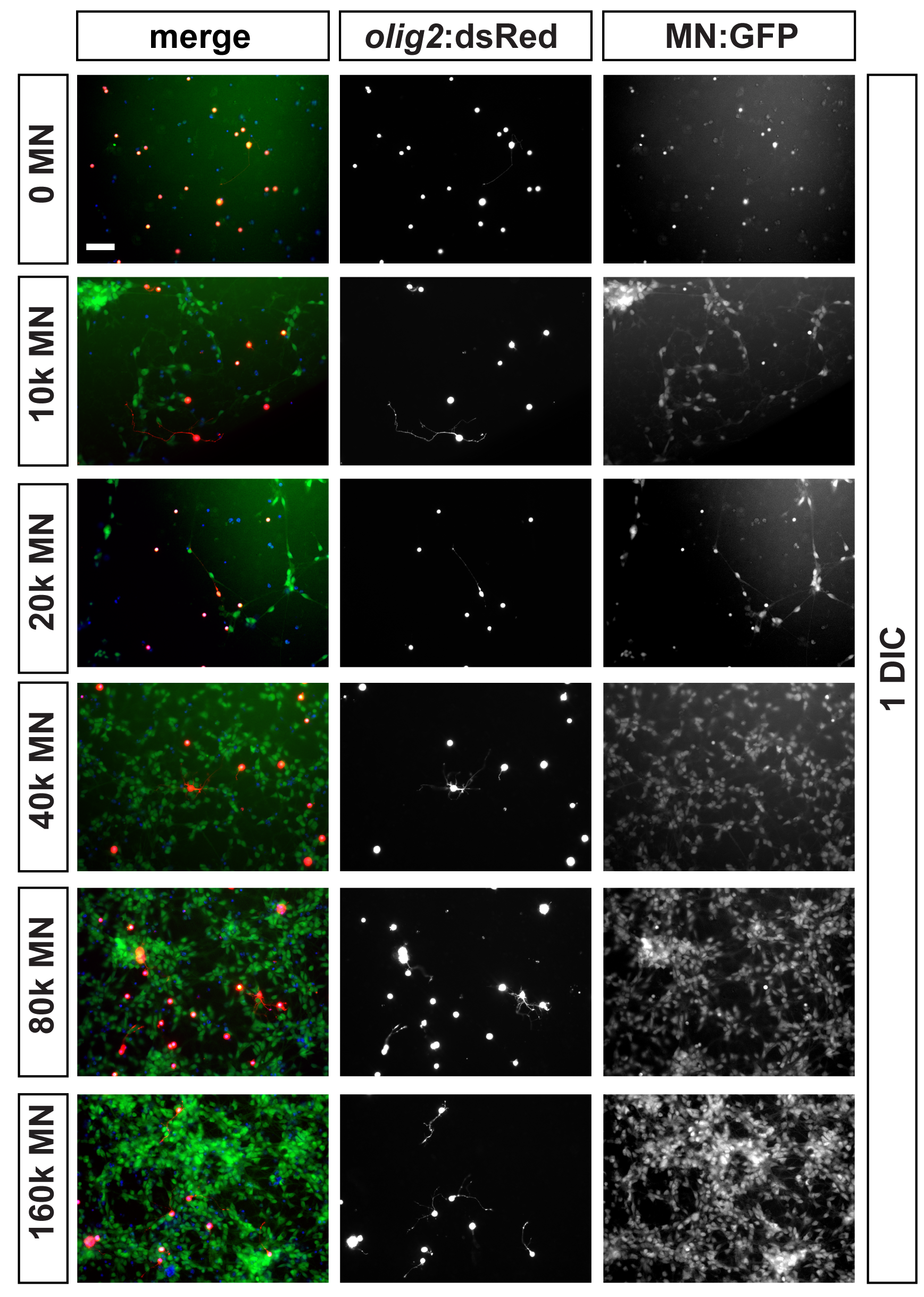

Supplement: FIGURE S3 — Morphology of OPCs varies with different numbers of MNs. Without the addition of MNs, most OPCs have a small and round morphology at 1 DIC. The number and branching complexity of OPC processes increase when MNs are added and are highest at the highest MN density of 160000 cells; scale bar represents 50 μm. [file Image_3.TIF]

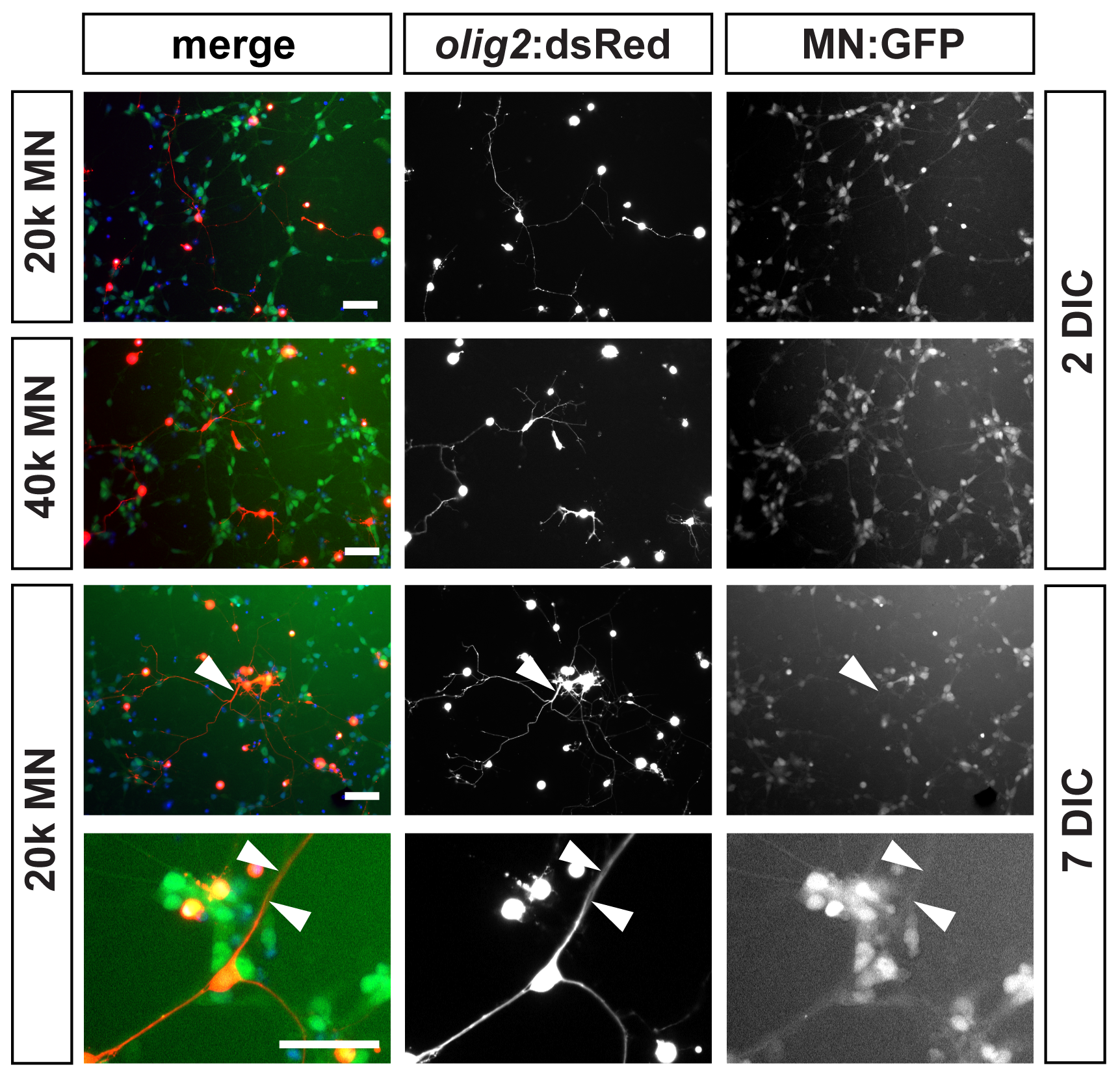

Supplement: FIGURE S4 — After longer culture periods OPCs adopt the morphology of mature OLs in OPC/MN co-cultures. At 7 DIC OPC processes increase in size, length and complexity of branching compared to 2 DIC. At 7 DIC the proximal region of the processes is enlarged compared to more distal regions (arrowhead). In the central part of some processes close association of axon and oligodendroglial processes can be seen (arrowheads); scale bar represents 50 μm. [file Image_4.TIF]
